# The impact of age on the risk of ipsilateral breast tumor recurrence after breast-conserving therapy in breast cancer patients with a > 5 mm margin treated without boost irradiation

**DOI:** 10.1186/s13014-019-1327-8

**Published:** 2019-07-10

**Authors:** Yuka Ono, Michio Yoshimura, Kimiko Hirata, Chikako Yamauchi, Masakazu Toi, Eiji Suzuki, Masahiro Takada, Masahiro Hiraoka, Takashi Mizowaki

**Affiliations:** 1 Department of Radiation Oncology and Image–Applied Therapy, Graduate School of Medicine, Kyoto University, 54 Shogoin Kawahara–cho, Sakyo–ku, Kyoto, Kyoto 606–8507 Japan; 2 Department of Radiation Oncology, Kyoto City Hospital, Kyoto, 604–8845 Japan; 3 Department of Radiation Oncology, Shiga General Hospital, Shiga, 524–8524 Japan; 4 Department of Breast Surgery, Graduate School of Medicine, Kyoto University, Kyoto, 606–8507 Japan; 5 Department of Radiation Oncology, Wakayama Red Cross Hospital, Wakayama, 640–8558 Japan

**Keywords:** Breast cancer, Breast-conserving therapy, Boost irradiation, Negative margin, Ipsilateral breast tumor recurrence, Age

## Abstract

**Background:**

The boost irradiation to the tumor bed following whole-breast irradiation (WBI) reduced the risk of ipsilateral breast tumor recurrence (IBTR). However, in Japan, almost all patients with a margin ≤5 mm receive boost irradiation to the tumor bed, but the decision to perform boost irradiation for those with a margin > 5 mm is dependent on the institution. Thus, institutional guidelines on utilizing boost irradiation for patients aged ≤40 or ≤ 50 years vary. We investigated the IBTR rate to assess the appropriate age for boost irradiation to the tumor bed with a margin > 5 mm.

**Methods:**

From January 1993 to December 2010, 419 patients with early-stage breast cancer and negative margins (> 5 mm) after breast-conserving surgery received WBI without boost irradiation. The Gray test was used to compare the cumulative incidence of IBTR among patients aged ≤40, 41–50, and ≥ 51 years. Hazard ratios were estimated using the Fine and Gray models. Furthermore, as a subgroup analysis, we investigated whether IBTR depended on the use of systemic therapy, such as anthracycline or taxane regimens.

**Results:**

The median follow-up time was 9.3 years. In multivariate analysis, only age predicted IBTR (*p* = 0.047). The 10-year IBTR rate was 15.7% in patients aged ≤40, 3.8% in those aged 41–50, and 2.0% in patients aged ≥51 years. The difference between patients aged ≤40 and 41–50 years was statistically significant (*p* = 0.045), whereas the difference between patients aged 41–50 and ≥ 51 years was not significant (*p* = 0.21).

**Conclusions:**

In our institutional surgical setting, when boost irradiation is performed only for patients with a margin ≤5 mm, the IBTR rate after WBI without boost irradiation was significantly higher in patients aged ≤40 years, suggesting that boost irradiation should be used for patients in this age group.

## Background

Breast-conserving therapy is a standard treatment for early breast cancer, as the Early Breast Cancer Trialists’ Collaborative Group (EBCTCG) has identified that radiotherapy after breast-conserving surgery (BCS) reduces the risk of recurrence and the risk of death from breast cancer [1, 2].

The European Organisation for Research and Treatment of Cancer (EORTC) phase III randomized trial explored the benefit of delivering a 16 Gy irradiation boost to the tumor bed after whole-breast irradiation (WBI) of 50 Gy in 5 weeks [3]. The analysis suggested that boost irradiation following WBI reduced the risk of ipsilateral breast tumor recurrence (IBTR) [3, 4]. In particular, the additional boost had a greater benefit in younger patients.

There have been several reports about the advantages of avoiding local recurrences. The EBCTCG has demonstrated that a low rate of local recurrence in the conserved breast after BCS could improve 15-year breast cancer survival [1]. Komoike et al. reported that young age was one of the important risk factors for local recurrence, and that the IBTR significantly correlated with subsequent distant metastases [5]. Furthermore, according to American Society for Radiation Oncology (ASTRO) consensus guidelines, boost irradiation should not be determined by the width of the surgical margin, in order to reduce the risk of IBTR in patients with negative margins of ‘no ink on tumor’ [6, 7]. In contrast, some disadvantages of boost irradiation have been identified, including the risk of moderate to severe fibrosis, cosmetic impairment, and the higher cost of an additional boost treatment [3, 4, 8].

Thus, ASTRO recommended tumor bed boost for patients aged ≤50 years with any grade of disease [9]. The 2017 St. Gallen international expert consensus conference on the primary therapy of early breast cancer concluded that radiation boost could be omitted in patients aged ≥60 years, patients with low grade tumors, or patients with a favorable biological profile [10]. Moreover, the National Comprehensive Cancer Network (NCCN) recommended the tumor bed boost in patients aged ≤50 years and patients with high-grade disease [11].

However, these recommendations were mainly based on the result of EORTC trial 22,881/10882, which was performed between 1989 and 1996 [12]. In the EORTC study, a negative margin was defined as no tumor cells on the ink; therefore, these recommendations may not be fully applicable to patients with a negative margin wider than 5 mm in Japan. Not all early breast cancer patients with a negative margin receive boost irradiation in Japan; on the contrary, less than half of institutions take young age into consideration when determining if boost irradiation is appropriate or not. Even when boost irradiation is added for young patients with a negative margin wider than 5 mm, institutional guidelines on whether to utilize boost for patients aged ≤40 or ≤ 50 years vary. Secondly, in our institution, systemic treatment for early breast cancer patients has been changed from oral pyrimidine fluoride agents to anthracycline-based regimens, taxane-based regimens, and trastuzumab. To the best of our knowledge, there are few reports evaluating IBTR in patients with a negative surgical margin wider than 5 mm and without radiation boost receiving modern systemic treatments because the use of boost irradiation is recommended based on the results of the EORTC study. Therefore, the main purpose of this retrospective cohort study was to investigate the rate of IBTR to assess the appropriate age for boost in the setting of a negative margin defined at > 5 mm and to clarify whether there is a potential benefit to a boost even when the margins are very wide. In addition, as a subgroup analysis, we assessed whether young age was also a risk factor for IBTR in patients receiving modern systemic treatments.

## Methods

### Patients

From January 1993 to December 2010, 620 patients were pathologically diagnosed with invasive carcinoma and treated with breast-conserving therapy at our institution. We excluded patients with pure ductal carcinoma in situ (DCIS), except for DCIS patients after biopsy or neoadjuvant chemotherapy. In addition, we excluded patients whose surgical margin was positive, close (≤5 mm), or unknown, and whose follow-up time was less than 1 year after BCS. The definitions of the surgical margin as negative or close were > 5 mm and ≤ 5 mm, respectively. We included patients who died or who suffered a recurrence less than 1 year after BCS. Thus, we retrospectively analyzed IBTR rates and prognostic factors in 419 early breast cancer patients with negative margins after BCS who were treated with WBI without boost irradiation. This study was approved by the institutional review board prior to initiation.

### Treatments

All patients were treated with partial resection and axillary lymph node dissection or sentinel lymph node biopsy. The tumors were resected with a 1–2 cm margin of macroscopically normal tissue.

WBI was performed using 2 tangential ^60^Cobalt or 4 or 6 MV photon beams. The regional lymph nodes were not irradiated deliberately. A median dose of 50 Gy (interquartile range (IQR): 50–50) was delivered.

Systemic therapy was selected by clinicians. The types of chemotherapy were as follows: oral pyrimidine fluoride agents (e.g. doxifluridine (5’DFUR), tegafur/uracil (UFT), or capecitabine), anthracycline-based regimens (e.g. CEF (cyclophosphamide/epirubicin/5-fluorouracil), AC (adriamycin/ cyclophosphamide), EC (epirubicin/cyclophosphamide), anthracycline plus taxane regimen (e.g. CEF plus docetaxel or paclitaxel, AC plus paclitaxel), taxane-based regimens (e.g. TC (docetaxel/cyclophosphamide)), and trastuzumab. Endocrine therapy consisted of selective estrogen receptor modulators, aromatase inhibitors, and luteinizing hormone-releasing hormone agonists, depending on patient menopausal status.

### Statistical analysis

The purpose of this study was to compare the IBTR rate among patients aged ≤40, 41–50, and ≥ 51 years. Furthermore, to investigate the influence of systemic therapy on IBTR, we analyzed IBTR in 2 periods: 1993–first half of 2003 (early period) and second half of 2003–2010 (later period).

IBTR was defined as a failure in the ipsilateral breast, which was determined by the breast surgeon and pathologist. The rates of IBTR and distant metastases were calculated from the date of the first BCS to the date of the event. The cumulative incidences of IBTR and distant metastases were estimated, with death before IBTR considered a competing risk. The Gray test was performed to compare cumulative incidences between the 2 groups. We estimated competing risk-adjusted hazard ratios (HRs) and 95% confidence intervals (95% CIs) using Fine and Gray models.

Overall survival (OS) was calculated from the date of BCS to the date of death from any cause, or the date of the last follow-up visit for living patients. The Kaplan–Meier method was used to estimate OS. The log-rank test was performed for OS comparisons. We estimated HRs and 95% CIs with Cox proportional hazard models for OS.

A chi-square test was performed to compare the characteristics of patients aged ≤40, 41–50, and ≥ 51 years. All of the following factors were studied: clinical T-stage (0, Tis, 1 or 2–4), pathological T-stage (0, Tis, 1 or 2–4), number of positive nodes (≤1 or ≥ 2), histological type of infiltrating carcinoma (ductal, lobular, or others), histological grade according to the Elston/Ellis modification of the Bloom–Richardson system (1, 2, 3, or unknown), hormone receptors (positive, negative, or unknown) and the use of chemotherapy or endocrine therapy. Multivariate logistic regression analysis was used to estimate the odds ratio of variables independently associated with outcomes. All statistical tests were two-sided, and the difference was considered statistically significant when the *P* value was < 0.05.

All statistical analysis were performed using EZR (Saitama Medical Center, Jichi Medical University, Saitama, Japan), which is a graphical user interface for R (the R Foundation for Statistical Computing, Vienna, Austria). Specifically, EZR is a modified version of the R commander designed to add statistical functions frequently used in biostatistics [13].

## Results

### Patient and tumor characteristics

The patient and tumor characteristics in the study cohort are summarized in Table 1. The median follow-up of patients from the date of the first BCS was 9.3 years (IQR: 6.8–12.9). The median age at diagnosis was 56 years (range, 26–85). In total, 43 (10.3%), 98 (23.4%), 124 (29.6%), and 154 (36.8%) of the patients were aged ≤40, 41–50, 51–60, and ≥ 61 years, respectively. Of these patients, 275 (65.6%) and 144 (34.4%) were diagnosed with pT0, Tis, 1 and pT2–4 disease, respectively. Particularly, there were 11 and 2 patients with pT0 and pTis, respectively. Two hundred and eighty-eight patients (68.7%) had no positive lymph nodes, 105 patients (25.1%) had 1–3 positive lymph nodes, and 26 patients (6.2%) had ≥4 positive lymph nodes.

**Table 1:** Tumor and treatment characteristics by age

	All (*n* = 419)		Age ≤ 40 years (*n* = 43)		Age41–50 years (*n* = 98)		Age51–60 years (*n* = 124)		Age ≥ 61 years (*n* = 154)		*P* value
cT stage											0.13
0, Tis, 1	194	46%	13	30%	44	45%	62	50%	75	49%	
2–4	225	54%	30	70%	54	55%	62	50%	79	51%	
pT stage											0.33
0, Tis, 1	275	66%	23	53%	68	69%	83	67%	101	66%	
2–4	144	34%	20	47%	30	31%	41	33%	53	34%	
Histology											0.14
IDC	381	91%	36	84%	91	93%	115	93%	139	90%	
ILC	8	2%	0	0%	3	3%	1	1%	4	3%	
Other	28	7%	7	16%	3	3%	7	6%	11	7%	
Unknown	2	0%	0	0%	1	1%	1	1%	0	0%	
Histological grade											N/A
1	68	16%	5	12%	11	11%	19	15%	33	21%	
2	123	29%	11	26%	22	22%	36	29%	54	35%	
3	72	17%	9	21%	13	13%	20	16%	30	20%	
Unknown	156	37%	18	42%	52	53%	49	40%	37	24%	
Number of positive nodes											N/A
0	288	69%	33	77%	58	59%	90	73%	107	70%	
1–3	105	25%	8	18%	35	36%	25	20%	37	24%	
≥ 4	26	6%	2	5%	5	5%	9	7%	10	7%	
Hormonal receptor status											N/A
Negative	110	26%	23	53%	26	27%	33	27%	28	18%	
Positive	296	71%	20	47%	69	70%	86	69%	121	79%	
Unknown	13	3%	0	0%	3	3%	5	4%	5	3%	
Chemotherapy											0.002
No	236	56%	19	44%	43	44%	73	59%	101	66%	
Yes	183	44%	24	56%	55	56%	51	41%	53	34%	
Endocrine therapy											0.31
No	115	27%	17	40%	27	28%	32	26%	39	25%	
Yes	304	73%	26	60%	71	72%	92	74%	115	75%	

*Abbreviations*: *cT* Clinical T, *pT* Pathological T, *IDC* Invasive ductal carcinoma, *ILC* Invasive lobular carcinoma, *N/A* Not applicable

### Relationship among tumor characteristics, treatment and age

The tumors and treatment characteristics according to age are shown in Table 1. Histological grade, hormone receptor status, and the use of chemotherapy as an adjuvant or neoadjuvant treatment were significantly different among the 3 groups stratified by age. The histological grade was unknown in more than 30% of patients in both groups.

### Relationship between ipsilateral breast tumor recurrence and age

**Fig. 1:**
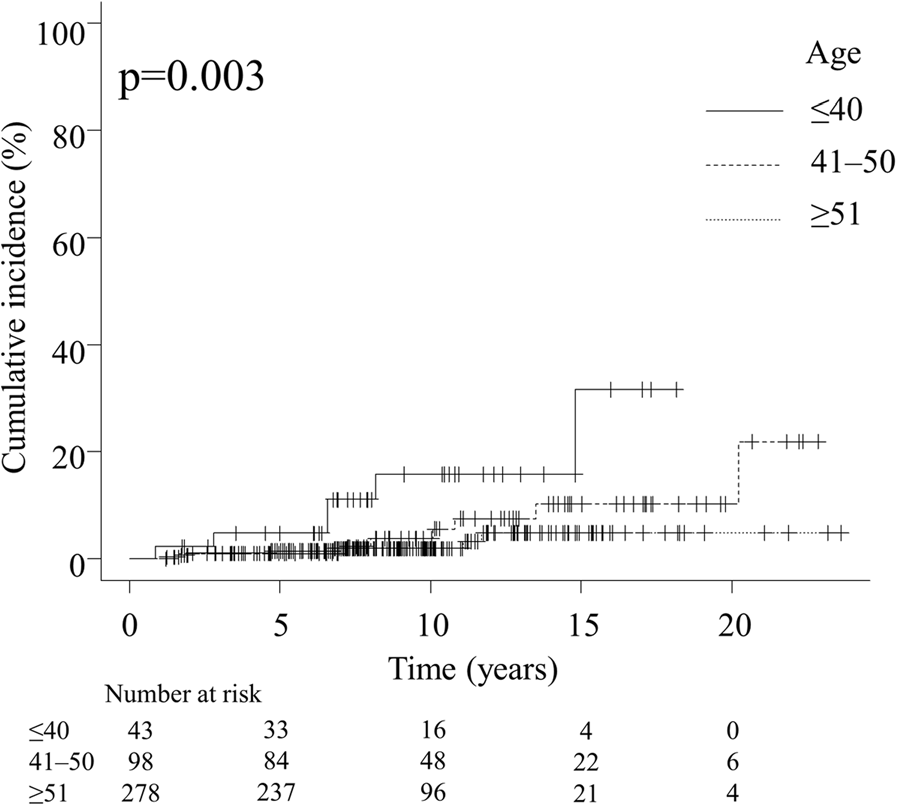
Cumulative incidence of ipsilateral breast tumor recurrence after whole-breast irradiation without boost irradiation by age

Younger patients had a significantly worse IBTR rate than older patients (Fig. 1). The cumulative incidences of IBTR at 10 years were 15.7% (95% CI 5.3–31.3) in patients aged ≤40 years, 3.8% (95% CI 1.0–9.9) in patients aged 41–50 years, and 2.0% (95% CI 0.7–4.3) in patients aged ≥51 years, respectively (*p* = 0.003). The difference between patients aged ≤40 and 41–50 years was statistically significant (*p* = 0.045), whereas that between patients aged 41–50 and ≥ 51 years was not significant (*p* = 0.21).

### Prognostic factors for ipsilateral breast tumor recurrence

The results of the Fine and Gray tests for the IBTR rate stratified by patient and tumor characteristics are shown in Table 2. The univariate analysis for the IBTR rate showed that being aged ≤40 and ≥ 41 years was significantly associated with the IBTR rate. The HR for IBTR was 4.36 (95% CI 1.67–11.39, *p* = 0.003) at 10 years. The univariate analysis for the IBTR rate also showed that being aged ≤50 and ≥ 51 years was significantly associated with the IBTR rate, and the HR was 2.97 (95% CI 1.19–7.42, *p* = 0.02). In the multivariate analysis, only age was a significant predictor of the IBTR rate; the HR was 2.73 (95% CI 1.01–7.40, *p* = 0.047).

**Table 2:** Univariate and multivariate models for ipsilateral breast tumor recurrence rate by patient and tumor characteristics

Factor	All patients *n* = 419					
	Univariate analysis			Multivariate analysis		
	HR	95% CI	*P* value	HR	95% CI	*P* value
Age in years						
≤ 40 vs. ≥41	4.36	1.67–11.39	0.003			
≤ 50 vs. ≥51	2.97	1.19–7.42	0.02	2.73	1.01–7.40	0.047
Pathological T stage						
0, Tis, 1 vs. 2–4	2.19	0.72–6.64	0.17	2.69	0.88–8.22	0.08
Histology						
IDC vs. others	0.93	0.23–3.84	0.92			
Histological grade						
1 vs. 2	0.72	0.25–2.13	0.56			
1 vs. 3	0.52	0.18–1.51	0.23			
Number of positive nodes						
0 vs. 1–3	0.96	0.52–1.79	0.91			
0 vs. ≥4	1.99	0.25–15.85	0.52			
Hormonal receptor status						
Negative vs. positive	1.09	0.67–1.77	0.74			
Chemotherapy						
No vs. yes	0.77	0.50–1.19	0.24	0.79	0.49–1.27	0.33
Endocrine therapy						
No vs. yes	0.71	0.24–2.12	0.54			

*Abbreviations*: *IDC* Invasive ductal carcinoma, *ILC* Invasive lobular carcinoma

### Subgroup analysis of IBTR in 2 periods: 1993–2003 (early period) and 2003–2010 (later period)

The selection of systemic therapy was determined by clinicians in both periods. Treatment characteristics in the early period (1993–2003) and later period (2003–2010) are shown in Table 3. In brief, in the later period, patients were less commonly treated with oral pyrimidine fluoride agents (6.6% vs. 36.8%), and more patients were treated with anthracycline or taxane regimens (43.4% vs. 3.6%) and trastuzumab (8.2% vs. 1.2%). The median follow-up of patients in the early and later periods was 14.4 years (IQR: 9.1–17.1) and 7.9 years (IQR: 6.5–10.2), respectively.

**Table 3:** Treatment characteristics by period

Characteristics	All		1993–2003		2003–2010	
	n	%	n	%	n	%
Chemotherapy						
None	236	56.3%	95	58.3%	141	55.1%
Oral pyrimidine fluoride agents	77	18.4%	60	36.8%	17	6.6%
Anthracycline-based regimen	26	6.2%	3	1.8%	23	9.0%
Anthracycline plus taxane regimen	54	12.9%	3	1.8%	51	19.9%
Taxane-based regimen	37	8.8%	0	0.0%	37	14.5%
Trastuzumab	23	5.5%	2	1.2%	21	8.2%
Others	4	1.0%	1	0.6%	3	1.2%
Endocrine therapy						
None	117	27.9%	61	37.4%	56	21.9%
SERM or AI	232	55.4%	88	54.0%	144	56.3%
SERM + LHRHa	61	14.6%	13	8.0%	48	18.8%
Others	9	2.1%	1	0.6%	8	3.1%

*Abbreviations*: *SERM* Selective estrogen receptor modulator, *AI* Aromatase inhibitor, *LHRHa* Luteinizing hormone-releasing hormone agonist

**Fig. 2:**
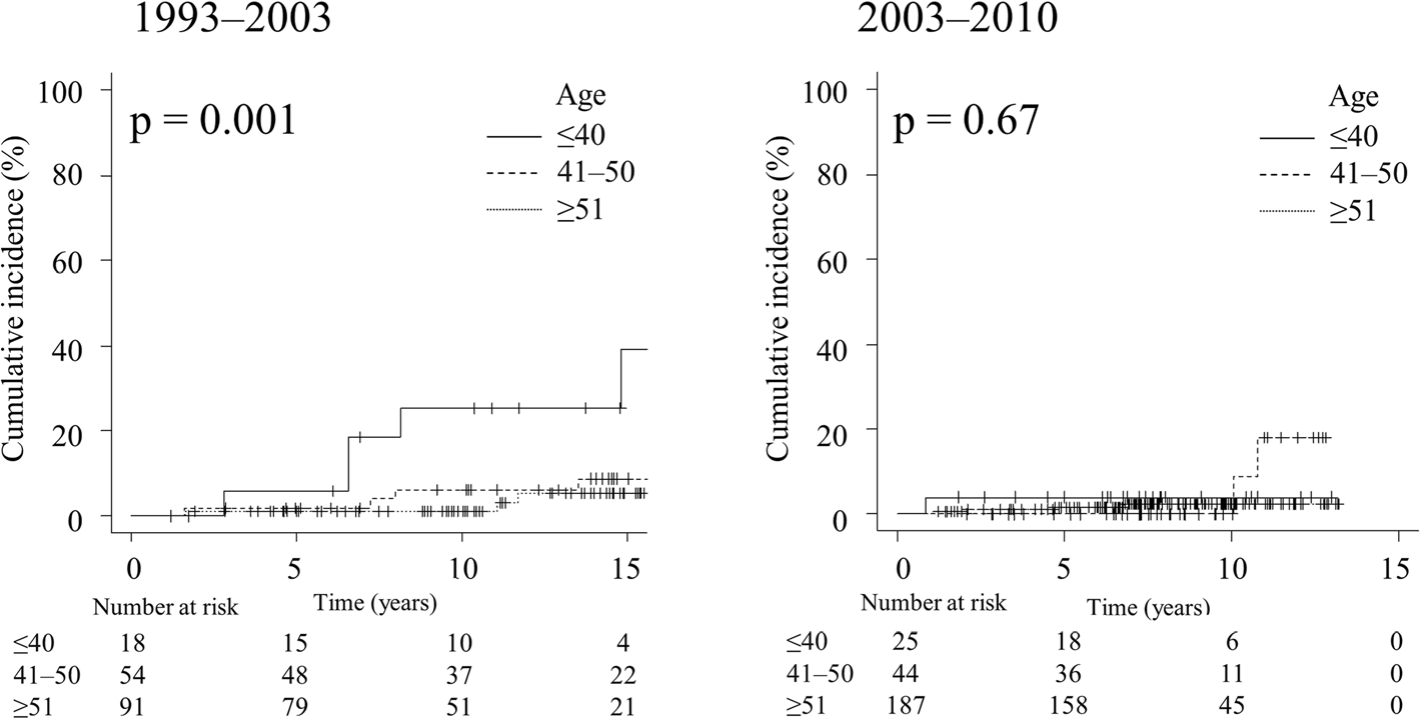
Cumulative incidence of ipsilateral breast tumor recurrence after whole-breast irradiation without boost irradiation by treatment period

Figure 2 shows that IBTR was correlated with patient age in the early period (*p* = 0.001). The difference between patients aged ≤40 and 41–50 years was significant (*p* = 0.02), whereas that between patients aged 41–50 and ≥ 51 years was not (*p* = 0.29). In contrast, in the later period, there was no statistically significant difference among patients aged ≤40, 41–50, and ≥ 51 years (*p* = 0.67).

Table 4 shows the IBTR rates stratified by age. Age groups were stratified in 4 groups so that the IBTR rate could be easily compared with that of the EORTC boost trial. In the early period, the IBTR rates in patients aged ≤40, 41–50, 51–60, and ≥ 61 years were 25.4, 6.2, 2.0, and 0%, respectively. In the later period, 8-year IBTR rates in patients aged ≤40, 41–50, 51–60, and ≥ 61 years were 4.0, 0.0, 2.8, and 2.3%, respectively.

**Table 4:** Ipsilateral tumor recurrence rate stratified by age in this cohort study and EORTC study

	**This cohort study**			**EORTC study**	
Age, years	All *n* = 419	1993–2003 *n* = 163	2003–2010 *n* = 256	No boost	Boost
Median f/u	9.3 y	14.4y	7.9y	10.8y	
	10y IBTR rate		8y IBTR rate	10y IBTR rate	
≤ 40	17.1%	25.4%	4.0%	34.9%	13.5%
41–50	3.7%	6.2%	0.0%	12.5%	8.7%
51–60	2.4%	2.0%	2.8%	7.8%	4.9%
≥ 61	1.6%	0.0%	2.3%	7.3%	3.8%
Total	4.0%	5.5%	2.2%	10.2%	6.2%

*Abbreviations*: *EORTC* European organisation for research and treatment of cancer, *f/u* Follow up time, *y* Years

### Distant metastases and overall survival

The 10-year cumulative incidences of distant metastases were 13.3% (95% CI 4.7–26.5) in patients aged ≤40 years, 17.2% (95% CI 10.0–26.1) in patients aged 41–50 years, and 8.1% (5.1–11.9%) in patients aged ≥51 (*p* = 0.26). The 10-year OS in patients aged ≤40, 41–50, and ≥ 51 years was 94.9% (95% CI 81.2–98.7), 92.0% (95% CI 82.8–96.4), and 94.1% (95% CI 90.1–96.5), respectively (*p* = 0.58). The 10-year cumulative incidence of distant metastases and the 10–year OS did not differ significantly among the 3 age groups.

## Discussion

Consistent with previous reports, this retrospective study from 1993 to 2010 showed that age was a prognostic factor for IBTR, although younger patients were treated with chemotherapy more frequently [2, 14–16]. For the patients with negative margin wider than 5 mm, there was a significant difference in the rate of the IBTR between patients aged ≤40 and 41–50 years; however, no significant difference was observed between patients aged 41–50 and ≥ 51 years.

Furthermore, the IBTR rate in this cohort study was much lower than that in the EORTC trial (Table 4) [3]. Even in the early period (1993–2003) when oral anti-cancer agents were mainly used both in our institution and the EORTC trial, the rate of IBTR in this study was lower than that of the no boost group in the EORTC trial. The patient and tumor characteristics were almost the same, except for the ratio of invasive ductal carcinoma. We postulate that the decreased IBTR rate could be due to differential definitions of negative margins, and potentially to differences in systemic treatments since 2003.

First, the definition of a negative margin was different between Japan and Europe/North America. The ASTRO consensus guideline and NCCN guideline define a negative margin as no tumor cells present on the ink [6–8] . In most Japanese institutions, the definition of a negative margin is a minimum distance between the tumor cells and the border of the resected specimen of > 5 mm. Meena et al. suggested that the use of no tumor cells on the ink as the standard for an adequate margin correlated with low rates of IBTR. Moreover, they evaluated the results of a meta-analysis on the relationship between specific margin widths (1 mm, 2 mm, 5 mm) and IBTR. Although the risk of IBTR decreased as the distance of negative margins increased (*p* = 0.058), the risk of IBTR was not significantly correlated with margin widths (*p* = 0.90) [6, 7]. Therefore, differential margin definitions could contribute to the decreased IBTR rate relative to the no boost group of the EORTC trial.

Next, we considered that the use of modern systemic treatments affected the low rate of IBTR in this study. The EORTC trial was performed from 1989 to 1996, and adjuvant systemic therapy were only given to patients with axillary lymph node involvement. Premenopausal patients received chemotherapy and postmenopausal patients received tamoxifen [17]. In contrast, as shown in Table 3, the regimens of systemic chemotherapy for early breast cancer in our hospital dramatically changed in 2003. From 1993 to 2003, oral pyrimidine fluoride agents were the therapies of choice; however, after 2003 anthracycline or taxane-based regimens and trastuzumab were used in our institution.

Among oral pyrimidine fluoride agents, 5’DFUR was the predominant treatment in this cohort. However, Tominaga et al. reported no significant differences in relapse-free and survival between adjuvant 6-month 5’DFUR monotherapy and surgery alone in early breast cancer [18]. UFT was the second most common anticancer agent. Park et al. found no statistically significant difference in 5–year disease-free survival and overall survival between UFT and CMF (C: cyclophosphamide, M: methotrexate, F: fluorouracil) [19]. CMF is the first regimen that reduces the breast cancer mortality rate as much as anthracycline plus cyclophosphamide [20]; however, CMF was inferior to anthracycline-based regimens, such as CAF and FEC (C: cyclophosphamide, A: doxorubicin (adriamycin), F: fluorouracil, E: epirubicin). The anthracycline plus taxane regimen further reduced the risk of breast cancer mortality [20]. Moreover, survival and recurrence rates were improved in patients receiving taxane-based regimens such as TC (T: docetaxel, C: cyclophosphamide) relative to AC (C: cyclophosphamide, A: doxorubicin (adriamycin)) [21]. Horton et al. revealed that trastuzumab reduces the odds ratio for local recurrence by approximately 50% in patients for whom WBI was administered in accordance with standard guidelines [22]. In our institution, Her2/neu status was not tested before year 2000, and trastuzumab was hardly used until 2006, and thus we could not quantify the impact of trastuzumab on the recurrence rates. As systemic chemotherapy has improved over time, the IBTR rate has significantly decreased. As a result, the IBTR rate among patients aged ≤40 years, 41–50 and ≥ 51 years does not differ significantly in the later period. However, we could not conclude that patients ≤40 years would not need an additional radiation boost in modern systemic treatment because the median follow-up of patients in the later period was only 7.9 years (IQR: 6.5–10.2).

Because this was a retrospective study, there are several inherent limitations. First, the median follow-up time was 9.3 years (IQR: 6.8–12.9), but the IBTR rate in patients aged 41–50 years increased after 10 years. Therefore, longer follow-up is needed. Moreover, we should clarify the risk of IBTR using a subgroup analysis of patients with local recurrence. As a result, more patients aged 41–50 years could benefit from additional boost irradiation. Second, only 43 (10.3%) patients were aged ≤40 years (41–50 years, 98 patients (23.4%); ≥51 years, 278 patients (66.3%)), and this could significantly influence the power of this analysis.

Third, in this study, no patients were treated with additional boost irradiation, so the effects of boost irradiation used in tandem with modern systemic treatment are unknown. A prospective randomized study to evaluate the effect of radiation boost to the tumor bed in modern systemic treatment is desirable. Finally, this result may not be applicable to all patients in other institutions. Because this was a retrospective cohort study at a single institution, the distance of the surgical margin and the definition of a negative margin are likely differ from those of other institutions. A multicenter trial would be beneficial to evaluate the use of boost irradiation in modern systemic treatment.

In the future, because of the development of systemic therapy, the rate of IBTR may decrease such that there will be no statistically significant difference among patients aged ≤40, 41–50, and ≥ 51 years. Further follow-up could assess the possibility of omitting radiation boost to the tumor bed even for early breast cancer patients aged ≤40 years with a > 5 mm margin after BCS and who have received appropriate modern systemic chemotherapy.

## Conclusions

In conclusion, this study showed that age was a prognostic factor for IBTR in women with a margin > 5 mm who were not given a tumor bed boost. In our institutional surgical setting, defining > 5 mm from the tumor as a negative margin, which is commonly used in Japan, the IBTR rate after WBI without radiation boost was significantly higher in patients aged ≤40 years. Cumulatively, these findings suggest that boost irradiation should be used to treat patients in this age group.

## Data Availability

The data that support the findings of this study are available from the corresponding author upon reasonable request.

## Abbreviations

5’DFUR: Doxifluridine
95% CIs: 95% confidence intervals
ASTRO: American Society for Radiation Oncology
BCS: Breast-conserving surgery
DCIS: Ductal carcinoma in situ
EBCTCG: Early Breast Cancer Trialists’ Collaborative Group
EORTC: European Organisation for Research and Treatment of Cancer
HR: Hazard ratio
IBTR: Ipsilateral breast tumor recurrence
IQR: Interquartile range
N/A: Not applicable
NCCN: National Comprehensive Cancer Network
OS: Overall survival
UFT: Tegafur/uracil
WBI: Whole-breast irradiation
